# Stimulation of adult hippocampal neurogenesis by physical exercise and enriched environment is disturbed in a CADASIL mouse model

**DOI:** 10.1038/srep45372

**Published:** 2017-03-27

**Authors:** C. Klein, S. Schreyer, F. E. Kohrs, P. Elhamoury, A. Pfeffer, T. Munder, B. Steiner

**Affiliations:** 1 Charité – University Medicine, Department of Neurology, Berlin, Germany

## Abstract

In the course of CADASIL (Cerebral Autosomal Dominant Arteriopathy with Subcortical Infarcts and Leukoencephalopathy), a dysregulated adult hippocampal neurogenesis has been suggested as a potential mechanism for early cognitive decline. Previous work has shown that mice overexpressing wild type Notch3 and mice overexpressing Notch3 with a CADASIL mutation display impaired cell proliferation and survival of newly born hippocampal neurons prior to vascular abnormalities. Here, we aimed to elucidate how the long-term survival of these newly generated neurons is regulated by Notch3. Knowing that adult neurogenesis can be robustly stimulated by physical exercise and environmental enrichment, we also investigated the influence of such stimuli as potential therapeutic instruments for a dysregulated hippocampal neurogenesis in the CADASIL mouse model. Therefore, young-adult female mice were housed in standard (STD), environmentally enriched (ENR) or running wheel cages (RUN) for either 28 days or 6 months. Mice overexpressing mutated Notch3 and developing CADASIL (TgN3^R169C^), and mice overexpressing wild type Notch3 (TgN3^WT^) were used. We found that neurogenic stimulation by RUN and ENR is apparently impaired in both transgenic lines. The finding suggests that a disturbed neurogenic process due to Notch3-dependent micromilieu changes might be one vascular-independent mechanism contributing to cognitive decline observed in CADASIL.

Cerebral Autosomal Dominant Arteriopathy with Subcortical Infarcts and Leukoencephalopathy (CADASIL) is the most common heritable cause of stroke and vascular dementia in adults^1^,^2^,^3^. It represents a genetic archetype of non-hypertensive ischemic cerebral small vessel disease. CADASIL patients carry dominant mutations in the *notch3* gene, which encodes a transmembrane receptor belonging to the Notch receptor family. Notch3 is required for the structural and functional integrity of small arteries. It is predominantly expressed in vascular smooth muscle cells and pericytes, controlling their arterial differentiation and maturation^4^,^5^. The highly stereotyped mutations alter the number of cysteine residues in the extracellular domain of Notch3 (Notch3^ECD^), leading to abnormal vascular accumulation of mutated Notch3^ECD ^3. In CADASIL, small and medium sized arteries characteristically exhibit pathognomonic deposits of granular osmiophilic material (GOM) containing mutated Notch3^ECD^. The resulting progressive degeneration of vascular smooth muscle cells (vSMC) leads to arteriole dysfunction, followed by subcortical lacunes with white matter injury. Cortico-cortical network disruptions in the frontal lobe have also been recently reported^6^. White matter infarcts are usually considered the leading cause of the progressive decline in cognitive function^7^. However, CADASIL patients show a decline in cognitive function prior to any infarcts^8^,^9^.

Interestingly, Notch3 has also been found to be expressed in neural precursor cells of the adult hippocampus^10^. Adult hippocampal neurogenesis is a lifelong process during which new neurons are generated in the subgranular zone (SGZ) and functionally integrated into neuronal networks^11^,^12^. This might represent a part of the CADASIL pathology as hippocampal neurogenesis has been demonstrated to play a crucial role in hippocampus-dependent learning and memory, maintaining cognitive flexibility during adulthood and ageing^13^,^14^,^15^. In general, Notch is a key regulator in the crosstalk between neurogenesis and angiogenesis. It controls vessel sprouting and is required for proliferation and differentiation of stem and precursor cells^16^,^17^,^18^. Moreover, adult hippocampal neurogenesis occurs in a highly vascularized niche of the SGZ^19^. Here, capillaries provide the supply of nutrients and oxygen to maintain the proliferative capacity of the stem and precursor cells. As *notch3* mutations in CADASIL lead to arteriole dysfunction and decreased blood flow^20^,^21^, it seems plausible that the resulting deficit in oxygen and glucose might influence adult hippocampal neurogenesis. Aside from the direct effect Notch3 can exert on neurogenesis by its expression in neural precursor cells, the Notch3-dependent vascular influence might, in turn, also be responsible for the observed cognitive impairments in CADASIL patients. Our previous study using a mouse model overexpressing Notch3 with a CADASIL mutation has demonstrated that adult hippocampal neurogenesis is indeed affected^22^. We have shown that neural cell proliferation and survival are reduced in the CADASIL mice at 12 months of age. This suggests functional consequences of the impaired neurogenesis on hippocampus-dependent learning and memory functions in the model and raises the question of whether physiological neurogenic stimuli might reverse the effect of the altered Notch3.

In the present study, we further elucidate how the short-term and long-term survival of newly generated neurons in the SGZ is regulated by Notch3, and how it depends on an intact Notch3 expression (Fig. 1). Adult neurogenesis can be robustly stimulated by physical exercise^23^ and environmental enrichment^24^. To investigate whether a dysregulated hippocampal neurogenesis can also be improved in CADASIL by these physiological neurogenic stimuli, female adult mice were housed in standard (STD), environmentally enriched (ENR) or running wheel cages (RUN) for either 28 days (short-term) or 6 months (long-term) (Fig. 1). To address these questions, the well-established transgenic mouse model overexpressing Notch3 with a CADASIL-causing point mutation (TgN3^R169C^) was used. To control for the effects of Notch3-overexpression in itself, mice overexpressing wild type Notch3 (TgN3^WT^), generated by the same approach as TgN3^R169C^, were used^25^.

## Results

### Notch3 overexpression results in reduced survival of newborn neurons after 6 months

In the long-term group, the noticeable but non-significant interaction of genotype and cage condition revealed that TgN3^WT^ mice displayed reduced BrdU+/NeuN+ cell numbers (Fig. 2f) under STD compared to WT and CADASIL mice (F(4,53) = 2.415, p = 0.06; post-hoc: TgN3^WT^ vs. WT, p < 0.01, TgN3^WT^ vs. TgN3^R169C^, p < 0.05). Such reduction in BrdU+/NeuN+ cell numbers under STD was not found in CADASIL mice (TgN3^R169C^ vs. WT, p > 0.05).

There were no changes in the number of neuronal cells in the short-term group in either transgenic mouse line under STD cage condition (WT vs. TgN3^WT^ and TgN3^R169C^, p > 0.05).

### Astrogliosis in CADASIL mice depends on the duration of cell survival

In the long-term group, CADASIL mice showed an increased percentage of newly generated BrdU+ cells differentiating into astrocytic S100β+ cells (F(2,59) = 4.030, p < 0.05; post-hoc: TgN3^R169C^ vs. WT, p < 0.05) (Fig. 3b). This was not seen in Notch3 overexpressing mice (TgN3^WT^ vs. WT, p > 0.05).

Astrogliosis did not occur in the short-term cell survival group in either transgenic mouse line (F(2,66) = 1.264, p > 0.05).

Representative images of the triple fluorescent staining for BrdU, S100β and NeuN are given in Fig. 4(a–h), exemplarily showing a co-labeled BrdU+/S100β+ cell (Fig. 4h) in the DG. Co-labeled BrdU+/NeuN+ cells are also presented (Fig. 4g).

### Neurogenic stimulation by short-term RUN or ENR is impaired in both Notch3 overexpressing and CADASIL mice

The significant interaction of both genotype and cage condition revealed that 28 days of RUN and ENR increased the number of BrdU+ cells (Fig. 2a) only in WT mice (F(4,60) = 4.495, p < 0.01; post-hoc: STD vs. RUN and ENR, p < 0.001) but not in in TgN3^WT^ or TgN3^R169C^ mice. Further cell characterization showed that this increase was due to an enhanced survival of BrdU+/S100β+ cells (F(4,60) = 6.037, p < 0.001; post-hoc: STD vs. RUN, p < 0.001) (Fig. 2c) and particularly of BrdU+/NeuN+ cells (F(4,60) = 4.147, p < 0.01; post-hoc: STD vs. RUN and ENR, p < 0.001) (Fig. 2e).

No such increase of (neuronal) cell numbers was found after 6 months of RUN or ENR (BrdU+ cells: F(4,53) = 2.108, p > 0.05; BrdU+/NeuN+ cells: F(4,53) = 2.415, p > 0.05) either in WT or transgenic mice (STD vs. RUN or ENR: p > 0.05).

Representative microscope images of the BrdU staining are given in Fig. 5, demonstrating that WT mice display more BrdU+ cells after 28 days of RUN (Fig. 5d) and ENR (Fig. 5g) compared to STD (Fig. 5a). Figure 5 also shows that RUN and ENR did not stimulate BrdU+ cell survival in TgN3^WT^ (Fig. 5b,e and h) or TgN3^R169C^ mice (Fig. 5c,f and i).

### Running wheel activity is reduced in CADASIL mice and age-dependently decreased in Notch3 overexpressing mice

During 28 days (Fig. 6a), TgN3^WT^ mice showed increased running wheel activity per 24 h compared to WT and CADASIL mice (F(2,543) = 84.66, p < 0.001; post-hoc: TgN3^WT^ vs. WT and TgN3^R169C^, p < 0.001). TgN3^R169C^ mice in turn ran a shorter distance per 24 h than WT (p < 0.001).

During 6 months (Fig. 6b), in contrast, running wheel activity was reduced in transgenic mice (F(2,1569) = 229.7, p < 0.001; post hoc: TgN3^WT^ and TgN3^R169C^ vs. WT, p < 0.001) with TgN3^R169C^ mice running even less than TgN3^WT^ mice (p < 0.001). Detailed analysis of running wheel activity over six months (Fig. 6c) revealed that the distance run per month decreased over time in TgN3^R169C^ mice (F(5,8) = 8.121, p < 0.01).

When considering just the first five days of RUN (Fig. 6d), which are most relevant for the stimulation of neural cell proliferation in the DG, WT and TgN3^WT^ mice covered similar distances, while TgN3^R169C^ mice showed significantly reduced physical activity per 24 h compared to WT and TgN3^WT^ mice (F(2,92) = 22.34, p < 0.001; post-hoc: p < 0.001).

### Motor coordination on the Rotarod is impaired in both transgenic mouse lines

TgN3^WT^ and TgN3^R169C^ mice spent significantly less time on the rotating rod than WT mice (F(2,16) = 6.309, p < 0.01; post-hoc: p < 0.05) (Fig. 6e). This indicates motor deficits in both transgenic mouse lines.

## Discussion

The present study aimed to investigate whether adult hippocampal neurogenesis in CADASIL can be influenced in short- and long-term by physiological stimuli, which have been shown to robustly enhance it in healthy animals and neuropathological disease models^26^,^27^,^28^,^29^. We found that the long-term survival of new neurons was reduced in Notch3 overexpressing but not CADASIL mice under STD cage conditions compared to WT. Moreover, short- and long-term neurogenic stimulation by RUN or ENR apparently failed in both transgenic mouse lines.

The decreased neurogenesis in Notch3 overexpressing mice of the long-term group replicates the finding of our previous study in six-months-old TgN3^WT^ mice^22^. The fact that the decreased neurogenesis already observed four weeks after BrdU cell labeling^22^ is still evident after five more months, shows that this is really due to a suppression of cell proliferation by Notch3 overexpression, as suggested in our previous work, rather than an influence on cell survival. Notch3 and Notch1 are possibly co-expressed in proliferating hippocampal precursor cells^22^. Moreover, Notch1 has been shown to be essential for progenitor pool maintenance and regulation of proliferation^16^,^18^,^30^. Therefore, it can be assumed that the suppression of cell proliferation by Notch3 is usually counteracted by Notch1-activated cell proliferation leading to a balanced cell proliferation rate. Overexpression of Notch3 clearly shifts the balance towards a down-regulation of precursor cell proliferation. Surprisingly, neurogenesis is not suppressed in TgN3^WT^ mice under short-term STD conditions. This might indicate an age-dependency of Notch3-dependent suppression of hippocampal neurogenesis with a counteracting mechanism being effective in younger mice of the short-term group but being lost during ageing in the long-term group.

In CADASIL mice, neurogenesis is not decreased as in Notch3 overexpressing mice. However, more newly generated cells differentiate into astrocytic cells in the long-term than in WT mice. As astrocytes are critical for neurogenesis and the neuronal long-term survival^31^, an increase in their portion here could represent a counteracting mechanism for a disturbed neurogenesis due to mutated and imbalanced Notch3. In support of this hypothesis, we also found an increased amount of astrocytic cells in WT animals induced by short-term RUN, which is similar to our previous findings showing different stimuli selectively affecting distinct subpopulation of newly generated hippocampal cells^32^. This may point towards the need for an intact microenvironment in the DG for a functional neurogenesis, as the generation of astrocytes is enhanced by RUN in parallel to neurogenesis.

Usually, RUN and ENR of short- or long-term durations are robust neurogenic stimulants in healthy or aged animals^33^,^34^ and in various rodent models of neuropathological diseases such as Alzheimer’s^29^ and Parkinson’s disease^26^. Here, RUN and ENR cage conditions increased hippocampal neurogenesis in healthy WT mice as expected. Although Notch3 overexpressing mice of the short-term group ran more in the running wheel than WT mice, despite a reduced motor coordination tested on the Rotarod, neurogenesis remains unaffected. This suggests that although neurogenesis is not yet reduced in younger TgN3^WT^ mice, it is already disturbed due to Notch3 overexpression as it could not be stimulated by RUN and ENR. In support of this, we have demonstrated in our previous work using a KCl-activation neurosphere assay that the proliferative activity of neural precursor cells was potentially reduced by Notch3 overexpression^22^. This might have prevented the activation by RUN or ENR. In contrast to the present results, Ables and colleagues^35^ have been able to restore neurogenesis by RUN in a Notch1 knock-out mouse. Knock-out of Notch1 specifically diminished the undifferentiated cell pool in the SGZ causing a decreased neurogenesis. RUN rescued the number of differentiated but not undifferentiated cells, indicating that this neurogenesis stimulation might not be mediated by Notch1. To clarify, if Notch3 may be involved instead, as suggested by the present results, a similar knock-out model but for Notch3 or a Notch3 antagonist^36^ could be used in follow-up studies.

In CADASIL mice, hippocampal neurogenesis was similarly not increased by RUN or ENR. In contrast to Notch3 overexpressing mice, CADASIL mice showed reduced physical activity in the running wheels throughout both durations. Motor coordination on the Rotarod was also impaired. This might be interpreted as the level of physical activity in RUN, and probably also in ENR, being insufficient to stimulate neurogenesis under this neuropathological condition. However, running wheel activity of less than 2000 m covered distance per day during 6 months has been shown to enhance neurogenesis^33^. In the present study, CADASIL mice ran mostly more than 2000 m per 24 h. Therefore, we suggest that the overexpression of mutated Notch3 disturbed the micromilieu of hippocampal precursor cells, which may have prevented these cells from reacting to RUN or ENR. This implies that not only functional Notch3 is crucial for the regulation of hippocampal neurogenesis but also its available amount in precursor cells itself and in the vascular neurogenic niche. This is of particular importance from a therapeutic point of view as it suggests that in CADASIL mutated Notch3 not only needs to be replaced but also the balance needs to be maintained.

Similar to CADASIL mice, Notch3 overexpressing mice also showed reduced physical activity (>2000 m distance covered) in the long-term and no stimulation of neurogenesis by RUN or ENR. But as they ran even more than WT mice in the short-term with still no change in neurogenesis levels, physical activity may not function as an adequate supportive therapy unless the amount of (functional) Notch3 is regulated at the same time. In ENR, however, physical activity is only one stimulation aside from visual, social and olfactory interaction with numerous other mice in a diversified equipped large cage. As the neurogenic stimulation by ENR was impaired to the same extent as by RUN, other functions than solely physical fitness could have been affected by mutated Notch3. Possible candidates are motivation, curiosity or anxiety, all of which might have been reduced in the transgenic mice, thus preventing the full experience of and benefit from ENR. This needs to be further investigated in these transgenic mouse lines.

In summary, we found that adult hippocampal neurogenesis *per se* is not altered in mice of the short-term group overexpressing wild type Notch3 or Notch3 with a CADASIL mutation. However, neurogenesis could not be stimulated by RUN or ENR of either duration, which may indicate a disturbed neurogenic process that is not reflected on the basal neurogenesis level. Considering this can be observed while no deficits in microcirculation or the vascular network have been reported^22^,^25^, it suggests an additional independent role of Notch3 in hippocampal function. We conclude that cell intrinsic deficits in Notch3 signaling contributing to changes in adult hippocampal neurogenesis by changing the micromilieu is one vascular-independent mechanism in CADASIL patients, which might be a supporting factor for the development of cognitive deficits.

## Methods

### Animals

Two different transgenic mouse lines were used in this experiment. TgN3^R169C^ mice carry the R169C point mutation at exon 4 of the *notch3* gene that causes cardinal pathological features of CADASIL^25^. TgN3^WT^ mice express wild type Notch3^25^. Both transgenic lines show a 4-fold overexpression of either the mutated or the wild type Notch3 transcript and protein^25^. The FVB/N background strain served as control. FVB/N mice were obtained from Janvier Labs (Le Genest-Saint-Isle, France). Transgenic mice were bred in the Research Institutes for Experimental Medicine of the Charité Berlin (FEM). All experiments were approved by the local animal ethics committee (Landesamt für Gesundheit und Soziales, Berlin) and were carried out in accordance with the European Communities Council Directive of 22 September 2010 (10/63/EU). The genotype was confirmed by PCR following tail biopsies (Primers: Notch3 forward: 5′ TTC AGTGGTGGCGGGCGTC 3′; Notch3 reverse: 5′ GCCTACAGGTGCCACCATTA CGGC 3′; Vector forward: 5′ AACAGGAAGAATCGCAACGTTAAT 3′; Vector reverse: 5′ AATGCA GCGA TCAACGCCTTCTC 3′). To minimalize stress and conflicts in the experimental groups, only females were included in the experiments. Water and rodent lab chow were provided *ad libitum* and a constant twelve hours light/dark cycle was applied.

### Experimental design

**Figure 1:**
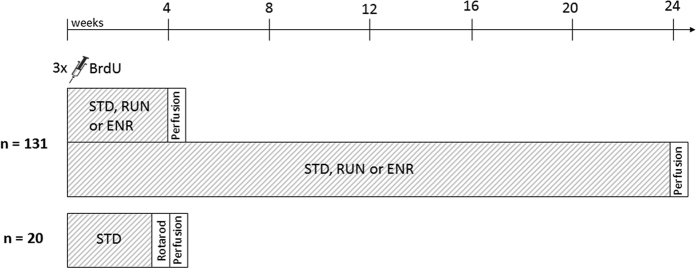
Experimental Design. Mice of each genotype were housed in standard (STD), running wheel (RUN) or enriched environment (ENR) cages for either a short (28 days) or a long duration (6 months).

**Figure 2:**
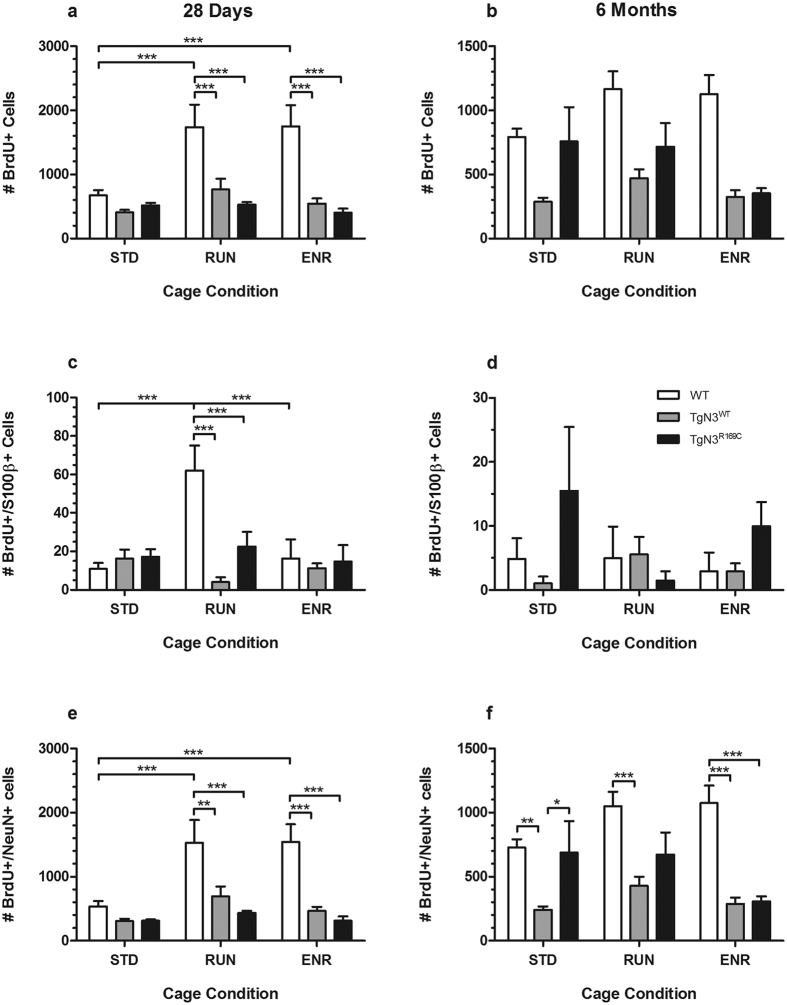
Results of the histological analysis of adult hippocampal neurogenesis in brain sections from WT, TgN3^WT^ and TgN3^R169C^ mice after 28 days (**a**,**c** and **e**) or 6 months (**b**,**d** and **f**) under standard (STD), running wheel (RUN) or environmentally enriched (ENR) cage conditions. The absolute number of BrdU+ (**a** and **b**), BrdU+/S100β+ (**c** and **d**) and BrdU+/NeuN+ cells (**e** and **f**) was quantified to determine the survival rate of proliferating cells, new astrocytic and new neuronal cells. New neuron survival is reduced in older (**f**) but not younger TgN3^WT^ mice (**e**). Neurogenic stimulation by RUN or ENR failed in both TgN3^WT^ and TgN3^R169C^ independent of the duration (**e** and **f**). Data are expressed as mean ± S.E.M. *p < 0.05, **p < 0.01, ***p < 0.001.

**Figure 3:**
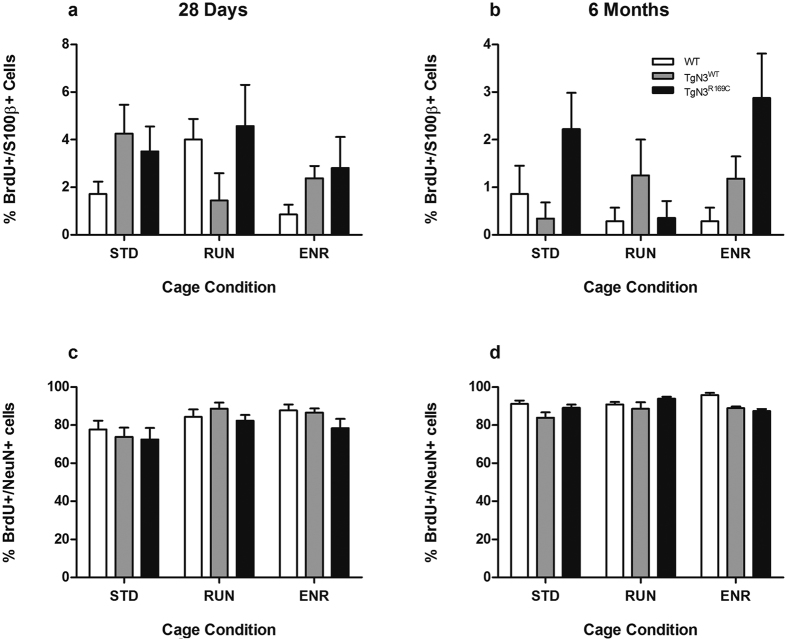
Results of the histological analysis of adult hippocampal neurogenesis in brain sections from WT, TgN3^WT^ and TgN3^R169C^ mice after 28 days (**a** and **c**) or 6 months (**b** and **d**) under standard (STD), running wheel (RUN) or environmentally enriched (ENR) cage conditions. The percentage of BrdU+/S100β+ (**a** and **b**) and BrdU+/NeuN+ cells (**c** and **d**) of all BrdU+ cells was determined to assess effects on the differentiation of BrdU+ cells to astrocytes and neurons. The percentage of BrdU+/S100β+ cells in TgN3^R169C^ is increased in older mice independent of RUN or ENR (**b**). Data are expressed as mean ± S.E.M.

131 eight to twelve week-old female FVB/N (WT), TgN3^R169C^ and TgN3^WT^ mice were each separated into three different cage conditions (Fig. 1). Mice maintained under standard conditions (STD) were housed in conventional cages (Makrolon cages, 0.27 m × 0.15 m × 0.42 m) in groups of two to five animals per cage. Mice kept in an enriched environment (ENR) were housed in groups of five to ten animals in larger cages (0.74 m × 0.3 m × 0.74 m), containing multiple plastic tubes, which varied in size and shape and were frequently rearranged, a cardbox house and a plastic house. In the third cage condition (RUN) mice were maintained in conventional cages in groups of two animals and provided with a running wheel (Tecniplast, Italy). Wheel turns were automatically recorded by LCD counters to monitor running wheel activity. Animals were kept in their specific cage condition either for a short (28 days) or a long (6 months) duration (Fig. 1). At the beginning of exposure to their specific cage condition, mice received three intraperitoneal (i.p.) injections of the mitotic marker Bromodeoxyuridine (BrdU, Sigma–Aldrich, Steinheim, Germany; 50 mg/kg in 0.9% NaCl) separated by an interval of 4 hours to label proliferating cells (Kuhn and Cooper-Kuhn, 2007) for the evaluation of their short- and long-term survival under the influence of wild type and mutated NOTCH3 overexpression as well as RUN and ENR.

A separate set of 20 eight to twelve week-old FVB/N, TgN3^R169C^ and TgN3^WT^ mice was exposed to the STD cage condition for 28 days and then tested on the Rotarod to assess motor coordination skills (Fig. 1)^37^.

### Rotarod

To test aspects of motor coordination, animals had to complete three consecutive trials on one day on the Rotarod (Columbus instruments, Columbus, OH, USA). The Rotarod consists of an elevated rod with modifiable rotating speed. Each mouse was placed on the rotating rod at a start speed of 5 rpm. When the animal found balance, the trial was started and the rod accelerated with a defined speed to a maximum of 65 rpm. The duration the animal could hold itself on the rotating rod was recorded automatically.

### Perfusion and Tissue Processing

All animals were killed at the end of the experiment. First, the mice were deeply anesthetized with Ketamine/Xylazine (10% Ketamine hydrochloride, WDT; 2% Rompun, Provet AG; i.p. injection) and then transcardially perfused using 0.1 M phosphate buffered saline (PBS) followed by 4% paraformaldehyde in PBS. Brains were removed and post-fixed overnight in PFA at 4 °C and afterwards transferred into 30% sucrose for 48 h for dehydration. Brains were then frozen in 2-methyl butane cooled with liquid nitrogen, and cut into 40 μm thick coronal sections (Bregma −0.22 mm to −3.80) using a cryostat (Leica CM 1850 UV).

### Immunohistochemistry and immunofluorescence

Adult hippocampal neurogenesis was evaluated by quantifying the number of proliferating cells, which were characterized by the incorporation of BrdU. Therefore, a one-in-six series of free-floating brain sections of each animal was pretreated with H_2_O_2_ and HCl and then incubated with a primary anti-rat BrdU antibody (AbD serotec, 1:500) overnight at 4 °C. The next day, the sections were incubated with a biotinylated secondary antibody (Dianova, 1:125), followed by streptavidin peroxidase complex (Vectastain Elite ABC Kit, Vector Laboratories). Antibodies were visualized by diamoniobenzidine (DAB)-nickel staining, after which the brain sections were mounted on microscope slides and coverslipped.

For a more detailed investigation of neuronal and astrocytic cell types, a triple fluorescent staining against BrdU, the specific endogenous marker for Neuronal Nuclei (NeuN) and the specific marker for mature astrocytes S100β was performed. Therefore, a one-in-six series of free-floating brain sections of each animal was pretreated with HCl, followed by an overnight incubation at 4 °C with primary rat anti-BrdU antibody (AbD serotec, 1:500), mouse anti-NeuN (Millipore, 1:1000) and rabbit anti-S100β (Abcam, 1:150). The next day, sections were incubated with fluorescent secondary antibodies RhodamineX (Dianova, anti-rat, 1:250), Alexa 647 (Dianova, anti-mouse, 1:300) and Alexa 488 (Invitrogen, anti-rabbit, 1:1000) for four hours. Finally, brain sections were mounted on microscope slides and coverslipped.

### Cell Quantification and image processing

For every animal, BrdU-positive (BrdU+) cells in the DAB staining were counted in nine sections containing the dentate gyrus (DG) with the SGZ, using a light microscope (Axioskop HB50/AC, Zeiss, Germany) and the 40× objective. Representative images of BrdU+ cells in the DG were taken using the 20× objective (Leica DMI 3000 B, bright field) and are shown in Fig. 5(a–i).

**Figure 4:**
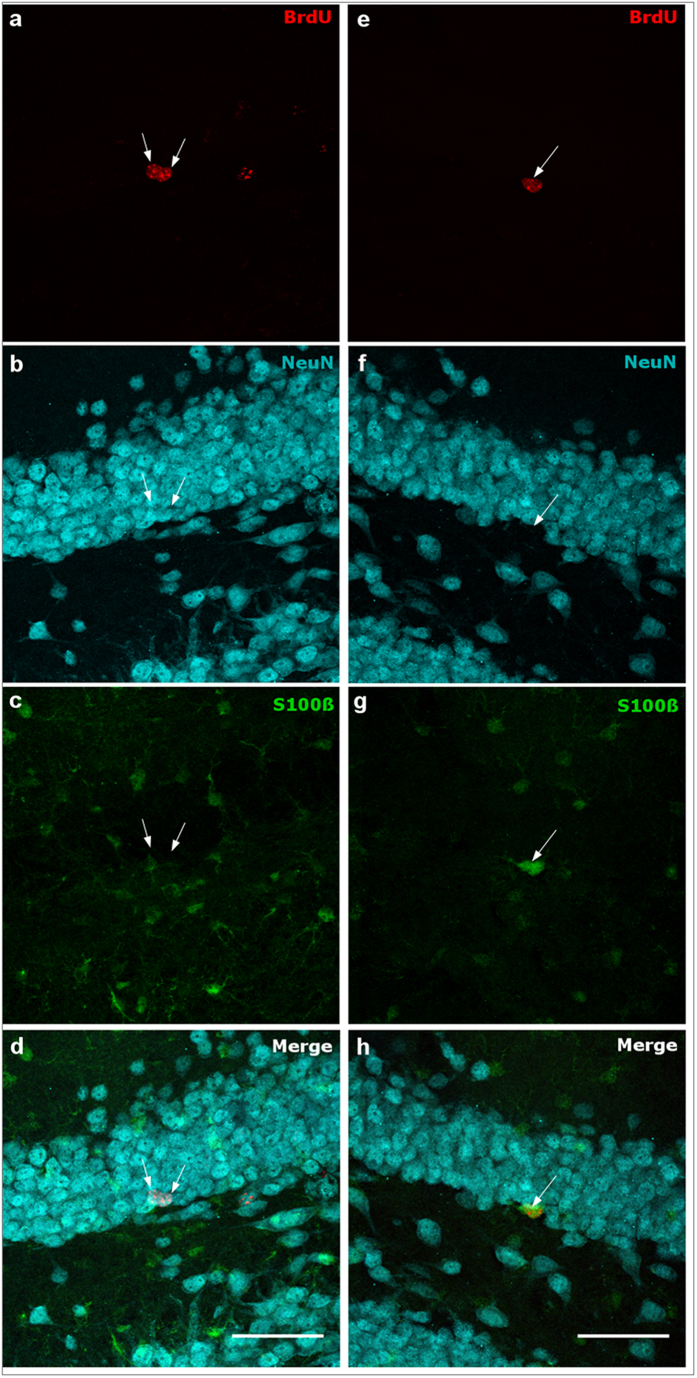
Representative confocal images of the triple fluorescent staining of the DG of two different mice (**a**–**d)**: TgN3^WT^ ENR 6 months; (**e**–**h**) TgN3^WT^ STD 28 days). Arrows point to BrdU+ cell nuclei (red, **a** and **e**), NeuN+ cell nuclei (cyan, **b** and **f**), S100β+ cells (green, **c** and **g**), two BrdU+/NeuN+ cell nuclei (**d**) and a BrdU+/S100β+ cell (**h**). Scale bar = 50 μm.

**Figure 5:**
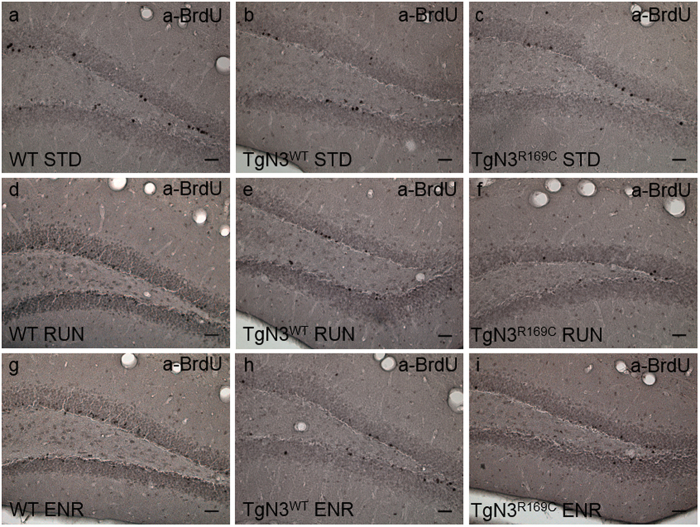
Representative light microscope images of the BrdU staining of the DG of each genotype after 28 days of STD (**a**–**c**), RUN (**d**–**f**) and ENR (**g**–**i**) cage conditions. They illustrate the increase in the number of BrdU-positive cells (black dots) in healthy WT animals after RUN (**d**) or ENR (**g**) and the missing stimulating effect on cell survival in Notch3 overexpressing (TgN3^WT^) and CADASIL mice (TgN3^R169C^). Scale bar = 100 μm.

**Figure 6:**
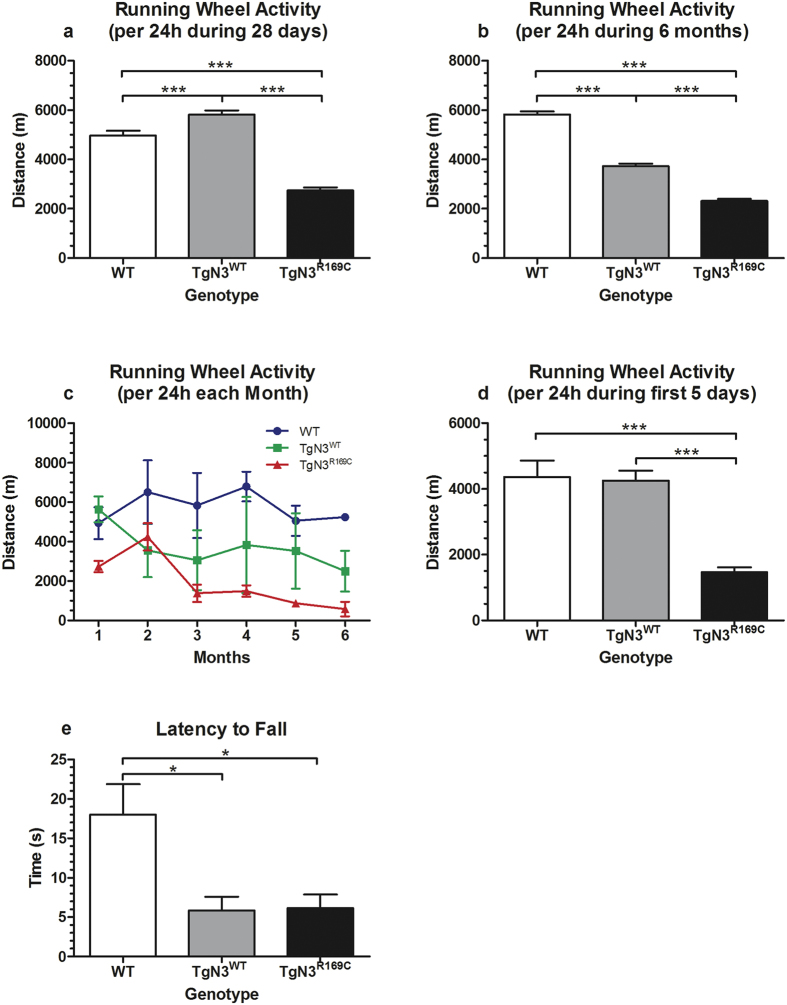
Effects of Notch3 overexpression (TgN3^WT^) and CADASIL (TgN3^R169C^) on physical activity per 24 h during 28 days (**a**), six months (**b** and **c**), during the first five days (**d**) under running wheel cage condition (RUN) and on Rotarod performance in transgenic mice corresponding to the 28 days group (**e**). Running wheel activity is reduced in TgN3^R169C^ mice and duration-dependently decreased in TgN3^WT^ mice (**a**–**d**). Motor coordination on the Rotarod is impaired in both transgenic mouse lines (**e**). Results of pairwise comparisons following a significant one-way ANOVA are displayed in the graphs. Data are expressed as mean ± S.E.M. *p < 0.05, ***p < 0.001.

To detect fluorescently co-labeled BrdU/NeuN-positive (BrdU+/NeuN+) and BrdU/S100β-positive (BrdU+/S100β+) cells, 50 BrdU+ cells spread across the rostrocaudal extent of the DG were sequentially scanned (z-stacks) using a confocal microscope (Leica DM 2500). The obtained ratio was used to determine the absolute cell number. Representative confocal images of the triple fluorescent staining are shown in Fig. 4(a–h). The confocal images were taken using the 40x oil immersion objective. To get a whole image of the examined cells, 19 sequentially taken images were z-stacked. The distance between the images was 0.34 μm. Fiji for Windows 32 was used to adjust brightness and contrast.

### Statistical analysis

The data sets of the short- and long-term group were graphically presented using GraphPad Prism 5 and separately analyzed using IBM SPSS Statistics 23. A two-way ANOVA was applied to analyze the effects of the investigated factors genotype and cage condition and their interaction on the numbers and percentages of BrdU+, BrdU+/NeuN+ and BrdU+/S100β+ cells. Running wheel activity during the short-term (28 days) and long-term (6 months) exercise intervention and Rotarod performance were analyzed by a one-way ANOVA. In case of a significant ANOVA, pairwise comparison using the Bonferroni post-hoc test was performed. The level of significance was set at p ≤ 0.05.

## Additional Information

**How to cite this article**: Klein, C. *et al*. Stimulation of adult hippocampal neurogenesis by physical exercise and enriched environment is disturbed in a CADASIL mouse model. *Sci. Rep.* **7**, 45372; doi: 10.1038/srep45372 (2017).

**Publisher's note:** Springer Nature remains neutral with regard to jurisdictional claims in published maps and institutional affiliations.
